# Pro-Inflammatory Effects of Inhaled Great Salt Lake Dust Particles

**DOI:** 10.21203/rs.3.rs-4650606/v1

**Published:** 2024-07-26

**Authors:** Jacob M. Cowley, Cassandra E. Deering-Rice, John G. Lamb, Erin G. Romero, Marysol Almestica-Roberts, Samantha N. Serna, Lili Sun, Kerry E. Kelly, Ross T. Whitaker, Jenna Cheminant, Alessandro Venosa, Christopher A. Reilly

**Affiliations:** University of Utah; University of Utah; University of Utah; University of Utah; University of Utah; University of Utah; University of Utah; University of Utah; University of Utah; University of Utah; University of Utah; University of Utah

**Keywords:** Great Salt Lake, Particulate Pollution, Pulmonary Inflammation, Transient receptor potential (TRP) channel, Climate Change, Dust, Toll-like receptor-4, Lipopolysaccharides, Transition Metal, Saline Lake

## Abstract

**Background:**

Climatological shifts and human activities have decimated lakes worldwide. Water in the Great Salt Lake, Utah, USA is at near record lows which has increased risks for exposure to windblown dust from dried lakebed sediments. Formal studies evaluating the health effects of inhaled Great Salt Lake dust (GSLD) have not been performed despite the belief that the dust is harmful. The objectives of this study were to illustrate windblown dust events, assess the impact of inhaled dust on the lungs, and to identify mechanisms that could contribute to the effects of GSLD in the lungs.

**Results:**

An animation, hourly particle and meteorological data, and images illustrate the impact of dust events on the Salt Lake Valley/Wasatch front airshed. Great Salt Lake sediment and PM_2.5_ contained metals, lipopolysaccharides, natural and anthropogenic chemicals, and bacteria. Inhalation and oropharyngeal delivery of PM_2.5_ triggered neutrophilia and the expression of mRNA for *Il6, Cxcl1, Cxcl2*, and *Muc5ac* in mouse lungs, was more potent than coal fly ash (CFA) PM_2.5_, and more cytotoxic to human airway epithelial cells (HBEC3-KT) *in vitro*. Induction of *IL6* and *IL8* was replicated *in vitro* using HBEC3-KT and THP-1 cells. For HBEC3-KT cells, *IL6* induction was variably attenuated by EGTA/ruthenium red, the TLR4 inhibitor TAK-242, and deferoxamine, while *IL8* was attenuated by EGTA/ruthenium red. Inhibition of mRNA induction by EGTA/ruthenium red suggested roles for transition metals, calcium, and calcium channels as mediators of the responses. Like CFA, GSLD and a similar dust from the Salton Sea in California, activated human TRPA1, M8, and V1. However, only inhibition of TRPV1, TRPV3, and a combination of both channels impacted cytokine mRNA induction in HBEC3-KT cells. Responses of THP1 cells were partially mediated by TLR4 as opposed to TRP channels and mice expressing a “humanized” form of TRPV1 exhibited greater neutrophilia when exposed to GSLD via inhalation.

**Conclusions:**

This study suggests that windblown dust from Great Salt Lake and similar lake sediments could pose a risk to humans via mechanisms including the activation of TRPV1/V3, TLR4, and possibly oxidative stress.

## Background

The Great Salt Lake (GSL) in Utah, USA is a terminal saline lake formed from the contraction of Lake Bonneville 10–20,000 years ago.(1, 2) The GSL is primarily fed by rivers originating in local mountains that now run through densely populated and industrialized areas. The GSL has no outlet causing the accumulation of minerals that give the lake its trademark salinity. Persistent anthropogenic pollutants also accumulate. While the extraction of minerals such as lithium, magnesium, and potassium sulfate from the GSL are of economic importance, GSL sediments also contain toxic metals such as iron, aluminum, copper, lead, arsenic, mercury and more.(3) This is attributable, in part, to accumulation from natural sources and being juxtaposed to one of the world’s largest copper mines.(4)

The GSL supports a complex ecosystem, harboring halophilic bacteria and archaea that are the foundation of a food web(5) supporting brine shrimp and fly populations that are an important food source for millions of migratory birds that visit the GSL annually.(6) The harvest and sale of brine shrimp eggs as food for aquaculture is also an important industry,(7) and between the extraction of minerals, brine shrimp eggs, and recreation, the GSL was estimated in 2012 to provide $1.32 billion in economic value per year.(8) Although maintenance of the GSL is of paramount importance for both environmental and economic reasons, it is in jeopardy due to climatological and human impacts on water input.

Exposed GSL lakebed sediment is a source of windblown dust which has become a major local public health concern because GSL sediment/dust is presumed to be harmful or “toxic.” Like the GSL, terminal saline lakes worldwide have been impacted by the effects of climate change and human activities including unsustainable irrigation practices.(9, 10) The Aral Sea saw a loss of 90% of its water volume from the 1960’s to the 2000’s,(11) leading to devastating health and economic impacts.(12, 13) Other examples include Owens Lake and the Salton Sea in California.(14–21) In the summer of 2022, the GSL reached historic lows, exposing expansive areas of dry lakebed that are prone to resuspension as respirable dust (GSLD) during high wind events. In 2020, the GSL playa was estimated to contribute ~ 23–34% of dust flux along the Wasatch front.(22, 23) In a viscous cycle, the GSLD also impacts snowpack, contributing to water loss. Local and national news stories repeatedly highlight the potential dangers of being exposed to GSLD, but no studies have evaluated the effects of inhalation (or other) exposure to these materials.

Exposure to particulate matter (PM) is linked to a variety of negative health effects. PM can originate from a multitude of sources including geological sources, mining, and combustion of coal, wood/biomass, and fossil fuels. PM derived from these different sources often vary in size, shape, and chemical composition and are likely to be distinct from PM arising from dried lakebeds of various nature. In general, the negative respiratory health outcomes associated with PM exposure involve interactions between PM, the lung epithelium, and other lung cells (e.g., macrophages, neurons) that sense the presence of a foreign substance and respond by triggering inflammation. However, the local and systemic effects can vary depending upon the dose, the duration of exposure, and the presence/absence of specific chemicals (e.g., metals, PAHs, etc.), pathogens, and physical properties (surface area, shape). PM_10_ and PM_2.5_ are a concern because they can enter and deposit throughout the respiratory tract and alveoli where components can become systemically distributed and where tissue damage is consequential for respiratory function. While the adverse effects and mechanisms associated with GSLD inhalation are unknown, studies from similar lakes suggest the potential for adverse effects.(9, 11, 12, 14, 17–19, 24)

The goals of this study were to illustrate the effects of windblown dust on local air quality, assess the impact of inhaled GSLD on the lungs, and to identify potential mechanisms underlying the effects of GSLD in the lungs.

## Methods and Materials

### Reagents and Chemicals

Allyl-isothiocyanate (AITC), menthol, HC-067047, icilin, N-(3-aminopropyl)-2-[(3-methylphenyl)methoxy]-N-(2-thienylmethyl)-benzamide hydrochloride (AMTB), and deferoxamine were purchased from Sigma-Aldrich (St. Louis, MO). A967079, nonivamide, TAK-242 (Resatorvid; Ethyl (6R)-6-[(2-chloro-4-fluorophenyl)sulfamoyl]cyclohexene-1-carboxylate), AMG-9810, and N-(1-((4-(2,4-dichlorophenyl)sulfonyl)amino-)-3-hydroxypropanoyl)-1-piperazinyl)carbonyl)-3-methylbutyl)-1-benzothiophene-2-carboxamide (GSK1016790A) were purchased from Cayman Chemical (Ann Arbor, MI). Drofenine hydrochloride was purchased from Santa Cruz Biotechnology (Dallas, TX). LJO-328 was provided by Dr. Jeewoo Lee, Seoul National University, and 2-(5-trifluoromethyl-pyridine-2ylsulfanyl)-1-(8-methyl-3,4-dihydro-2H-quinolin-1-yl)-ethanone (abbreviated as 007) was synthesized as previously described.(25)

### GSLD, Coal Fly Ash (CFA), and Salton Sea Dust (SSD):

GSL sediment was collected in July 2022 from the following locations near the Great Saltair, Great Salt Lake State Park, and Kennecott Copper mine/refinery and tailings pond on the southeast shore of the GSL: 40.74741717055637, −112.19173244734307; 40.75102960965917, −112.19785977880764; 40.76006570075401, −112.1951335289701; 40.76119114860347, −112.19305240202357; 40.758499910134354, −112.18936958930033. Material was collected from the top ~ 1” of sediment and ranged in consistency and moisture content. Some samples were a mix of grey, brown, and black sand while others were black and tar-like or spongy and brown. The materials were dried for 10 days at 40°C in the laboratory and 100 g of each sample was combined and subjected to resuspension using compressed filtered air in a 4L Erlenmeyer flask from which suspended PM was pulled through a 10-stage Andersen cascade impactor (1L/min; Thermo Andersen Inc., Smyrna GA). Impactor stages were weighed before and after to obtain the mass of each particle size fraction and PM < 3.1 μm was pooled to generate the GSLD PM_2.5_ used for this work. A similar PM, CFA, used for comparison, was collected from the Hunter power plant in Castledale, UT and screened to ≤ 10 μm. CFA composition and its effects on TRP channels and lungs of mice have been previously described.(26–28) For inhalation studies CFA PM_2.5_ was prepared as above. Salton Sea sediments were collected in March 2024 from the following locations: 33.38616, −115.53463; 33.28608, −115.53497; 33.28584, −115.53506; 33.47647, −115.89211; 33.34564, −115.73151; and 33.34535, −115.73265. The materials varied from a muddy/sandy material to a typical grey/black beach sand. SSD PM_2.5_ was also prepared from a pool of these samples as above.

### Dust Event Modeling and Characterization

PM data were collected from a network of low-cost sensors deployed across the Salt Lake valley/Wasatch front. Readings from April 18, 2022-April 24, 2022, were used to construct an animation depicting real-time PM_10_ (an accepted marker of dusts as opposed to typical anthropogenic PM) in the Salt Lake Valley/Wasatch front airshed. This analysis used measurements from 438 low-cost particulate matter sensors (from the University of Utah, AIrUs, and PurpleAir, PAIIs) and Federal Reference/Equivalence Measurements (FRMs/FEMs) from the Utah Division of Air Quality (UDAQ). The low-cost sensor measurements were corrected using correction factors (CFs) developed by co-locating the AirU and PAIIs at UDAQ’s Hawthorne and Rose Park monitoring stations. These measurements were screened for outliers, corrected using the co-located correction factors, and were incorporated into a Gaussian Process (GP) regression model, which included customized kernel functions that incorporate distance, time and elevation to obtain continuous-valued spatio-temporal estimates of PM concentration throughout study region, complete with a confidence value describing the accuracy of the measurement. This model allowed the construction of dense PM colormaps, as shown in the animation. The performance of the PM sensors, the infrastructure, and the accuracy of the PM_2.5_ estimates have been previously demonstrated.(29–33) PM_10_ concentrations are estimated using the ratio of PM_2.5_ to PM_10_ concentration from the UDAQ’s Hawthorne monitoring station using the method described in Kaur and Kelly.(34)

PM_2.5_ and PM_10_ data, as well as accompanying meteorological data were also obtained from local air quality monitoring stations operated by the UDAQ (https://air.utah.gov/dataarchive/index.htm). A second date range (April 16–19, 2023) surrounded a separate dust event on April 18, 2023, triggered by an approaching storm. Data for this event are from the following monitoring stations: Harrisville (41.3028593, −111.9874424), Herriman (40.4950126, −112.0347781), Lindon (40.3387775, −111.7152311), the Utah Technical Center (40.7746306, −111.9471611), and Hawthorne Elementary School (40.7544692, −111.8734927) in Salt Lake City, UT.

Images illustrating an event on March 2–3, 2024, were taken from the corresponding author’s office window at the University of Utah on March 1 and 2, 2024 using an iPhone SE.

### GSLD Characterization

Lipopolysaccharides/endotoxin (LPS) associated with GSLD PM_2.5_ was quantified using the Pierce^™^ Chromogenic Endotoxin Quant Kit from Thermo Fisher Scientific. Bacterial growth from GSLD PM_2.5_ was evaluated by dispersing PM onto salt-fortified (0, 5% and 12%) yeast peptone agar plates and incubating for 1 week at 37°C. Colonies were picked and subjected to colony PCR and sequencing of the 16S RNA amplicons obtained using the 27F and 1492R primers to identify bacteria. Results are provided in Additional File 1. Metal analysis was performed by the University of Utah Iron and Heme Core on an Agilent 7900 inductively coupled plasma mass spectrometry (ICP-MS) instrument operated in He collision mode. GSLD PM_2.5_ (5 mg) was placed in 500 μL HNO_3_ + 100 μL H_2_O_2_, incubated overnight, and 300 μL heated to dryness at 98°C. The solution was then incubated overnight in 2 mL 2% HNO_3_ containing 100 ppb Ge internal standard. The supernatant was diluted 10x and 100x with 1x Ge solution to a final volume of 2 mL. Samples were run in triplicate and results from the 10x and 100x dilutions were averaged. The physical and elemental characteristics of GSLD were also evaluated using scanning electron microscopy energy-dispersive spectroscopy (SEM-EDS) using an FEI Quanta 600 FEG scanning electron microscope with energy dispersive X-ray spectroscopy. Electron microscopy was performed at the University of Utah Electron Microscopy Core Laboratory. The Organic/elemental carbon content of GSLD was assayed by Sunset Laboratories (Cary, NC). Briefly, ~ 10 mg of PM_2.5_ was deposited quartz filters mounted in a conical filter/concentrator under constant flow (~ 1L/min) and sent to Sunset Laboratories for analysis.

### Discovery Level Liquid Chromatography-Tandem Mass Spectrometry (LC/MS/MS) Analysis of GSLD Sediments and PM _2.5_:

GSLD associated chemicals were “extracted” from 100 mg GSLD sediment and PM_2.5_ using water and ethanol, dried under air, and assayed using electrospray LC/MS/MS on a Thermo Vanquish Flex UPLC system interfaced with an LTQ Velos Pro linear ion trap mass spectrometer. Data interrogation/spectral matching was performed using the Global Natural Products Social (GNPS) Molecular Networking Knowledge Base(35), as previously described.(36) Chromatographic fractionation was achieved using a BEH C_18_ column (150 × 3 mm i.d.; 1.7 μm particle size; Waters, Milford, MA) at 50°C with gradient elution. The mobile phases were (A) 0.1% formic acid in H_2_O and (B) methanol and a flow rate of 100 μL/min was used. The percentage of B varied as follows: 2% B at 0 min, 2%–100% (0 to 50 min), hold at 100% (50 to 55 min), 100%–2% (55.0 to 55.1 min), and hold at 2% (55.1 to 60 min). The mass spectrometer was programmed to either positive or negative ions m/z 100–750 (in separate acquisitions) using the “double play” data-dependent MS/MS mode. MS/MS scans were triggered by analytes having a signal intensity set at ~ 5x baseline using a collision energy of 35%. The top 3 peaks of each scan were analyzed, and dynamic exclusion was active: The parameters were repeat count = 3; repeat duration = 5 s; exclusion list size = 25; and exclusion duration = 5 s. Data were analyzed using GNPS. The .raw files were converted to mzML files using MS Convert, uploaded into GNPS and processed using the Library Search feature. Default criteria were used with the exception that the minimum matched peaks = 4 and top hits per spectrum = 5. A cosine score of 0.85 was used as a cut-off criterion for including chemicals in Additional File 2.

### Mice

Studies were approved by the University of Utah IACUC committee. Male and female C57Bl/6 mice (8–10 weeks of age) were used. The mice were housed (5/cage) and maintained in an AALAC accredited vivarium and fed and watered *ad lib* with standard chow. Mice were either exposed to GSLD PM_2.5_ by inhalation using an inExpose system (Scireq, Quebec Canada) or via oropharyngeal (OPA) aspiration using a total volume of 25 μL suspended PM_2.5_. For inhalation, mice were exposed to an atmosphere of ~ 50 ± 5 μg/m^3^ GSLD PM_2.5_ for 15 min intervals (1x, 2x, 3x in 1 day, or 6x over 2 days) to model short-term exposure to peak concentrations of PM_2.5_ during major dust events. GSLD PM_2.5_ suspensions were generated using a PALAS RBG 1000ID operated at 1,200 RPM with a piston feed rate of 200 mm/h. The bias airflow was 2L/min and the PM concentration was monitored in real time using a Casella CEL-712 Microdust Pro (1/chamber) immediately prior to the exposure chambers. Inhaled doses of ~ 27 ng (1.1 μg/kg) were estimated for a 15 min exposure using equations 1 and 3 from Borghi *et al*.(37) and averaged mouse tidal volume and minute volume values in Schwarte *et al*.(38) Applying deposition estimates of ~ 4%, ~ 12.5% and ~ 50% for the deep lung/alveolar, tracheobronchial, and nasal/oropharyngeal regions,(39) deposited doses of ~ 1, ~3, and ~ 13 ng of PM were estimated. Of note, ICP-MS analysis of lung tissue revealed > 2-fold changes in sodium, aluminum, strontium, lead, lithium and barium following 6x exposure, all of which are associated with GSLD PM_2.5_. For OPA delivery, the doses were 0 (saline only), 0.1, 0.5, 2.5, and 10 mg/kg (0, 2.5, 12.5, 62.5, 250 μg/dose). This dose range encompasses those used in mouse acute particle toxicity studies utilizing oropharyngeal delivery to model moderate to high-dose human exposures.(40–45) In all cases, 24 h after exposure, mice were sacrificed by Euthasol injection. Exposure to CFA PM_2.5_ was also performed as above using the 3x exposure paradigm.

### Generation of the Trpv1^N606D^ “humanized” mice:

CRISPR Cas9 reagents were designed by the University of Utah Mutation Generation and Detection Core. The sgRNA N20 sequence used was 5’-GTAGTGACACTGATCGAGGA-3’ and the single-stranded oligo deoxyribonucleic acid (DNA) nucleotide (ssODN) HDR donor sequence was 5’-cacccacacctctttctcttgcgacctgtagCCGTAGTGACcCTGATtGAGGATGGGAAGAATgACTCACTGCCTGTGGAGTCCCCACCACACAAGTGTC-3’. Nucleotide changes are in lower case. The ssODN contained stabilizing 5’ and 3’ phosphorothioate modifications, a single base change to introduce the N606D mutation and two silent changes to block CRISPR cutting after HDR and to introduce a unique restriction enzyme site for genotyping (EcoN1). See Additional File 3. The University of Utah Transgenic & Gene Targeting Mouse Core Facility co-electroporated a ribonucleoprotein complex and the ssODN donor molecule into single cell embryos harvested at day 0.5. Electroporated embryos were rinsed and surgically implanted into oviducts of 0.5-day pseudopregnant females. Founders were genotyped with simple PCR (F primer: 5’-AGTGGCTTTCCTGCTGAGGG-3’; R primer: 5’-AACTCCAGGTCACCCATGCC-3’) and EcoN1 restriction enzyme digestion. Founders with insertion were bred and the resulting N1 mice were sequenced to confirm correctness. All protocols followed AALAC procedures and were approved by the University of Utah IACUC committee.

### Bronchoalveolar Lavage (BAL) and Differential Cell Counting

BAL was collected from mice by inserting a cannula through a small incision in the trachea. Cold saline (1 mL) was slowly infused into the lungs, first adding 0.5 mL, retracting, then adding the entire 1 mL. This first 1 mL of BAL fluid was immediately placed on ice, clarified by centrifugation, and the supernatant frozen at −80°C for protein analysis. An additional 4 mL of BAL fluid was collected 1 mL at a time and immediately placed on ice. The cells from the initial 1 mL and 4 mL sample were then pooled, concentrated by centrifugation, resuspended in 1 mL cold saline, counted, and 5,000 cells affixed to slides using a cytospin. Cells were stained with Giemsa for manual differential cell counts.

Differential cell counting was also evaluated by tissue flow cytometry, using the antibody staining protocol and gating as described by Nguyen *et al*.(46) Briefly, following lavage, lungs were cleared of blood by cardiac perfusion with saline solution, removed from the chest cavity, minced, and transferred into a 50 mL conical tube and incubated (37°C, 30 min) in DMEM + 5% FBS + 2 mg/ml Collagenase D (Roche, Indianapolis, Indiana). Digested lungs were then passed through 70-μm nylon mesh to obtain a single-cell suspension, counted and mixed with ACK Lysis Buffer (Thermo Fisher Scientific) to remove remaining red blood cells. The BAL and tissue cell pellets (1M cells) were resuspended in 100 μl staining buffer (PBS + 0.1% sodium azide) and incubated with anti-mouse CD16/32 antibody for 10 min at 4°C to block nonspecific binding. This was followed by 30 min incubation with fluorescently tagged antibodies or appropriate isotype controls (0.25–1.5 μg/10^6^ cells) for 30 min (4°C). Cells were then spun and resuspended in staining buffer for viability staining (30 min at 4°C) followed by fixation in 2% paraformaldehyde. Analysis was performed on a Cytek Aurora flow cytometer (Cytek Biosciences, Fremont, California) using a gating strategy of singlet, viable, CD45 + cells as described by Nguyen et al.(46) Data analysis was performed using FlowJo software (FlowJo, LLC, Ashland, Oregon).

### Histological Analysis

Following BAL collection and perfusion, the lungs were inflated with 10% neutral buffered formalin at 20 cm H_2_O fixed pressure for 5 min. The cannula was then removed, and the trachea tied closed while maintaining inflation. The lungs and trachea were then removed and placed in 10% neutral buffered formalin for 48 h, dissected to obtain tissue from the hilum to the lower portion of the left lobe, rinsed with (2%) sucrose and dehydrated in 70% ethanol. Lungs were embedded in paraffin, and serially sectioned (5 μm thick) followed by staining with Eosin and Hematoxylin by ARUP Laboratories (Salt Lake City, UT).

### RNA Purification and Cytokine mRNA Expression

Before fixation, the left bronchus was tied closed and the left lobe of the lung removed and placed in RNALater solution at 4°C. Within 1 week, the lungs were homogenized in Trizol Reagent (1 mL/50–100 mg of tissue) and phase separated with chloroform. The RNA was then precipitated with ethanol followed by RNA purification using the PureLink RNA Mini Kit (Invitrogen; Carlsbad, CA). RNA was stored at −80°C until used. RNA (2 μg) was reverse transcribed using the ABI High-Capacity cDNA Kit + RNase Inhibitor (Applied Biosystems, Foster City, CA). Cytokine gene expression was analyzed by quantitative real-time polymerase reaction using a Life Technologies QuantStudio 6 Flex instrument and TaqMan probes (Applied Biosystems) for mouse *Il6* (Mm00446190_m1), *Cxcl1* (Mm04207460_m1), *Cxcl2* (Mm00436450_m1), and *Muc5ac* (Mm01276718_m1). Values for relative gene expression were normalized to the housekeeping gene mouse glyceraldehyde-3-phosphate dehydrogenase (Mm99999915_g1), which exhibited stability across treatments utilizing the comparative cycle threshold (ΔΔCT) method.

### Cell Culture:

Cells were maintained in a humidified cell culture incubator at 37°C with a 95% air: 5% CO_2_ atmosphere. Immortalized human bronchial epithelial (HBEC3-KT) cells (ATCC; Rockville, MD) were grown in Airway Epithelial Basal Medium supplemented with Bronchial Epithelial Cell Growth Kit (ATCC; Rockville, MD). HEK-293 cells (ATCC; Rockville, MD) stably overexpressing the ultrasensitive fluorescent sensor protein GCaMP6s were cultured in DMEM:F12 media containing 5% fetal bovine serum and 1x penicillin/streptomycin. THP-1 cells (ATCC; Rockville, MD) were maintained in RPMI 1640 media supplemented with 10% fetal bovine serum and 0.05 mM 2-mercaptoethanol at 0.3–1M cells/mL. For experiments, cells were plated and differentiated for 72h using phorbol 12-myristate acetate (25 nM).

### *In vitro* Cytotoxicity

HBEK3-KT cells were plated at ~ 10k/well in a 96 well plate in Airway Epithelial Cell Basal Medium. After 24 h at 80–90% confluence, the media was removed and replaced with 200 μL of media containing PM. Cells were treated with GSLD and an elementally similar PM, CFA at concentrations ranging from 0 to 10 mg/mL (i.e., 0–6.25 mg/cm^2^). After 24 h, cell viability was determined using the Dojindo CCK8 Reagent (8% v/v) by incubating cells for 2 h at 37°C. To avoid particle interference, the plate was briefly centrifuged and the clarified supernatant transferred to a new plate for measuring the absorbance at 490 nm. Results were normalized to cells treated with media only. THP1 cells were plated and differentiated at 120k/well in a 96 well growth media supplemented with 25 μM PMA and 1x penicillin/streptomycin. Cells were treated with GSLD ranging from 0 to 1.125 mg/mL (i.e., 0–0.53 mg/cm^2^). In all cases, treatment solutions were prepared in media at the highest concentration, sonicated for 30 min, and vortex mixed for 1 min followed by serial dilution in Eppendorf tubes with 1 min of vortex mixing between each dilution step.

### Human TRP Channel Activation Assays

Calcium flux assays were conducted in HEK-293 GCaMP6s cells transiently transfected with the TRPA1, M8, truncated ΔM801 TRPM8, TRPV1, TRPV3, and TRPV4 expression plasmids in 96-well plates (coated with 1% gelatin) 48h prior to assay, as previously described.(26, 40, 41, 46, 47) Thirty minutes prior to analysis, the media was replaced with LHC-9 containing 1 mM probenecid and 0.75 mM trypan red (ATT Bioquest). Changes in fluorescence were captured on an EVOS FL Auto Imaging System (Life Technologies) and treatment-induced changes in cellular fluorescence were quantified from fluorescence micrographs. All agonist/particle treatment solutions were prepared in LHC-9 at 3x concentration and added to cells at room temperature. Activation studies used a final PM concentration of 2.3 mg/mL (180 μg/cm^2^). All data were normalized to the maximum attainable change in fluorescence elicited by ionomycin (10 μM).

### *IL6/8* mRNA Expression Studies

HBEC3-KT were plated in 12-well plates at a density of 25k/cm^2^. After 72 h, the media was aspirated and replaced with 1 mL of media containing GSLD. Treatments were prepared by suspending GSLD PM_2.5_ in media, sonicating for 20 min, vortex mixing for 1 min, and briefly vortex mixing immediately prior to applying to cells to ensure a homogenous suspension of the GSLD. For these studies a concentration of 0.25 mg/mL or 66 μg/cm^2^ was used. For pathway inhibitor studies, cells were pre-treated for 30 min followed by co-treatment with GSLD. At the desired time-point, the media was aspirated and stored. Cells were then washed with PBS and frozen (−80°C). RNA was isolated using the PureLink RNA Mini Kit. Cytokine mRNA quantification was achieved as above using TaqMan probes for human *IL6* (Hs00174131_m1) and *IL8* (Hs00174103_m1). Values for relative gene expression were normalized to the housekeeping gene human *beta-2-microglobulin* (Hs00187842_m1), which exhibited stability across treatments utilizing the comparative cycle threshold (ΔΔCT) method.

### Statistics

Results were analyzed using a combination of t-tests, 1- and 2-way ANOVA with post-testing using either a Dunnett’s or Bonforroni test, as specified in figure legends.

## Results

### Wind events increase dust (PM _2.5/10_) along the Wasatch front:

**Figure 1:**
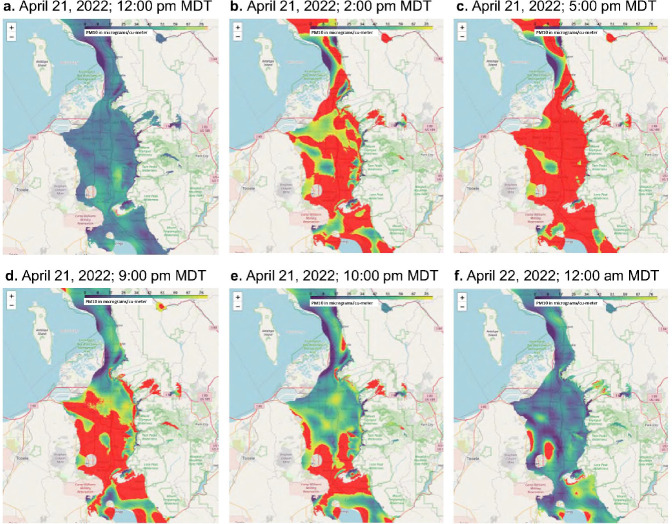
Time coded images of a dust event on April 21, 2022. (a-f) Snapshots were taken from Additional File 4 (movie). Purple represents PM_10_ concentrations 0–10 μg/m^3^ and red represents ≥100μg/m^3^.

**Figure 2:**
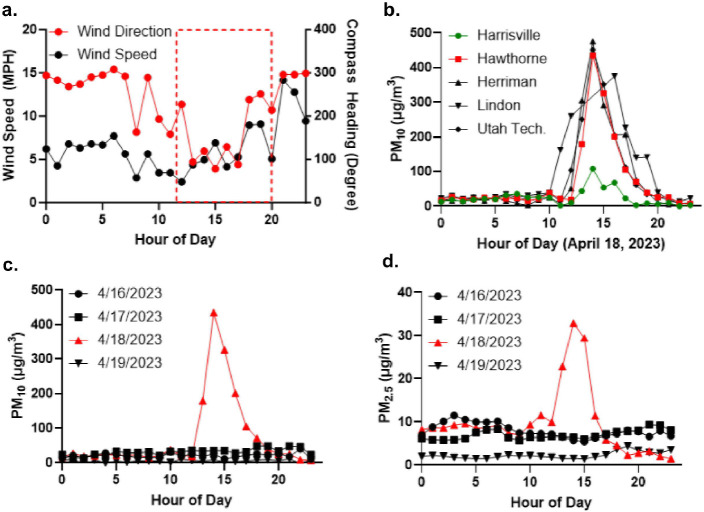
Meteorological conditions and PM_2.5/10_ measurements from a dust event on April 18, 2023. (a) Wind speed (left y-axis, black line) and direction (right y-axis, red line). Red box indicates the period when PM readings were highest, prior to rain starting. (b-d) Hourly PM_10_ and PM_2.5_ readings from state monitoring stations from different locations in the Salt Lake Valley/along the Wasatch front. The red line is the Hawthorne monitoring station in downtown Salt Lake City and the green line for Harrisville, north of Salt Lake City.

**Figure 3:**
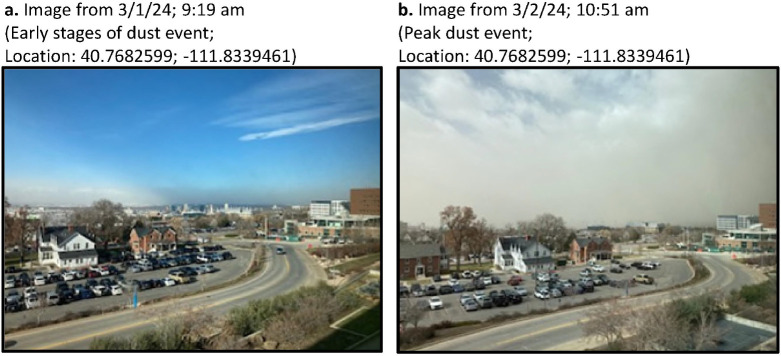
Images of a dust event on March 2–3, 2024. Images were taken from the corresponding author’s office window on the University of Utah campus, looking west towards Salt Lake City and the south end of the Great Salt Lake.

Fig. 1 shows screenshots from an animation (Additional File 4) illustrating the distribution of PM_10_ in the Salt Lake Valley/Wasatch front surrounding a dust event on April 21, 2022. Hourly PM_2.5_ data from state-run air quality monitoring stations is shown for 2022, April 2022, and 4/1922–4/22/22 in Supplemental Fig. 1 (Additional File 4). Similarly, on April 18, 2023, winds shifted from east (~ 100°) to west-northwest (300°) after ~ 3:00 pm MDT (Fig. 2a) increased PM_2.5_ and PM_10_ in the air along the Wasatch Front/in the Salt Lake Valley (Fig. 2b–d). As in the 2022 event, hourly PM_2.5_ and PM_10_ concentrations were low (< 10–20 μg/m^3^) on April 16, 17, and 19, 2023, but spiked to ~ 35 ± 7 and ~ 400 ± 40 μg/m^3^. A local news story describing the event was titled “*Blowing dust causes dirty rain to fall across northern Utah*.”(48) Of significance, the PM_2.5_ and PM_10_ concentrations measured at the Harrisville monitoring station, which is north of Salt Lake City, and partially sheltered from west-northwest winds by Promontory Point, exhibited smaller increases in PM (PM_10_ ~ 108 μg/m^3^; Fig. 2b). Images of a similar dust event occurring on March 2–3, 2024, are shown in Fig. 3. While the precise contribution of GSLD was not determined, dust originating from past and current areas of dried lake sediments likely contributed to the overall dust burden.

### GSLD contained metals, organic materials/chemicals including LPS, and was non-sterile

**Figure 4:**
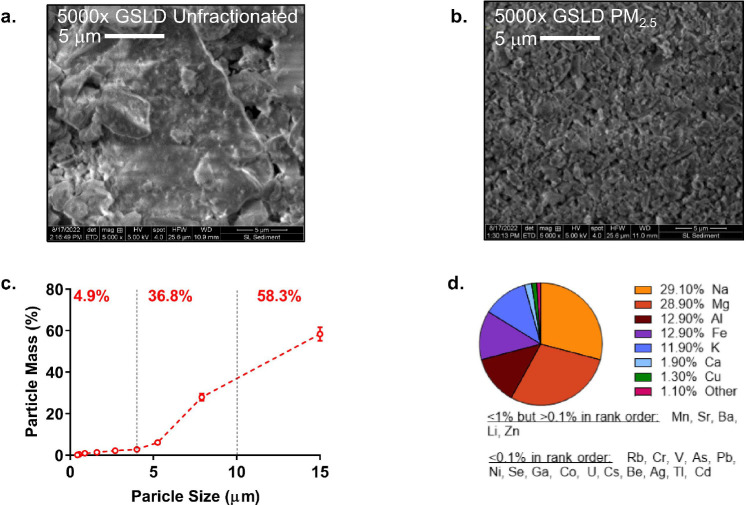
Characteristics of GSL sediment and PM_2.5_. (a) 5,000x electron micrograph of unfractionated GSL sediment and (b) GSLD PM_2.5_. (c) Percentage of particle size fractions by mass from resuspension of GSL sediments in the laboratory. Data points represent the different stages of the Anderson Cascade Impactor, where the x-value of 15 represents the fraction present in the pre-separation stage. (d) Elemental composition of GSLD PM_2.5_ using ICP-MS. Major constituents (>1%) are represented as a pie chart with those <1% and 0.1% listed in rank order below the pie chart.

Electron micrographs of unfractionated GSLD showed grey sheet-like materials varying in size (Fig. 4a), while PM < 3.1 μm (i.e., the GSLD PM_2.5_ used in the majority of our studies), was a light grey powder with similar irregularly shaped particles (Fig. 4b). PM < 3.1 μm represented ~ 5% of total mass recovered during resuspension of the sediments in the laboratory, whereas PM 3.1–10 μm was ~ 36.8%, consistent with the relative abundances of PM_2.5_ and PM_10_ during dust events.

ICP-MS analysis of GSLD sediment and GSLD PM_2.5_ revealed an abundance of sodium, magnesium, aluminum, iron, potassium, calcium, and copper (Fig. 3d and Supplemental Fig. 2). GSLD sediment and PM_2.5_ also contained metals known to be toxic including manganese, lead, arsenic, cadmium, uranium, etc., albeit at concentrations < 1%. Enrichment (> 2-fold) in the PM_2.5_ sample was observed for beryllium, gold, cadmium, copper, strontium, uranium, and selenium, whereas lead was ~ 2-fold lower. Of note, the ICP-MS analysis was limited in that only the elements sufficiently leached from the PM using HNO_3_ + H_2_O_2_ treatment were analyzed. However, a similar, albeit less detailed elemental composition was found using SEM-EDS (Supplemental Figs. 3 and 4).

GSLD PM_2.5_ was 97.9 ± 0.6% organic carbon. Organic species included LPS (90 ± 2 EU/mg) and numerous chemicals preliminarily identified in pooled aqueous and ethanol extracts using LC/MS/MS analysis and GNPS database searching (Additional File 2). Finally, GSLD PM_2.5_ was not sterile and harbored multiple bacteria that were cultured and preliminarily identified by 16s sequencing *including Rossellomorea vietnamensis, Virgibacillus dokdenensis, Bacillus pakistanensis, bacillus haikouensis, Thalassobacillus cyri, Thalassobacillus devorans, Crenalkalicoccus roseus, Methyloglobulus morosus, Bacillus zhangzhouensis, Bacillus safensis, Streptococcus pyogenes, Mesobacillus subterraneus, Mesobacillus boroniphilus* (Additional File 3). A more comprehensive study of GSLD has identified hundreds of novel taxa.(49)

### GSLD PM_2.5_ inhalation and OPA caused acute lung inflammation in mice

**Figure 5:**
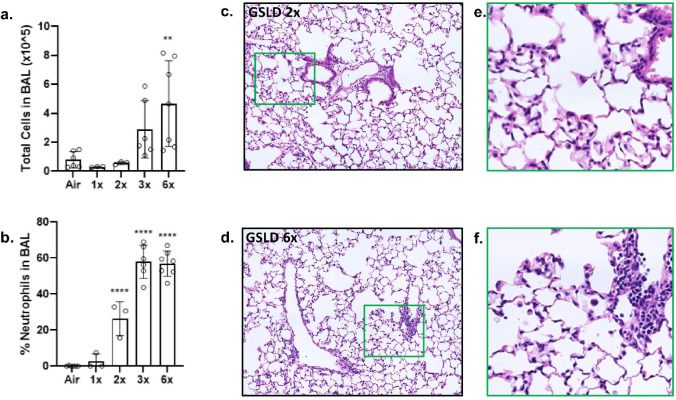
Effect of GSLD PM_2.5_ inhalation dose and duration on BAL cell counts. (a) Total cell counts and (b) percentage of neutrophils in BAL following inhalation exposure of mice to GSLD PM_2.5_ (50 mg/m^3^) for 15 min (1x), 30 min (2x), or 45 min (3x) over the course of one day or 90 min over 2 days (6x). (c-d) 40x photomicrographs and (e-f) expanded areas of interest of hematoxylin and eosin-stained lung tissue from mice exposed 2x or 6x to GSLD PM_2.5_. **p<0.01 and ****p<0.0001 using 1-way ANOVA and Dunnett post-test.

**Figure 6:**
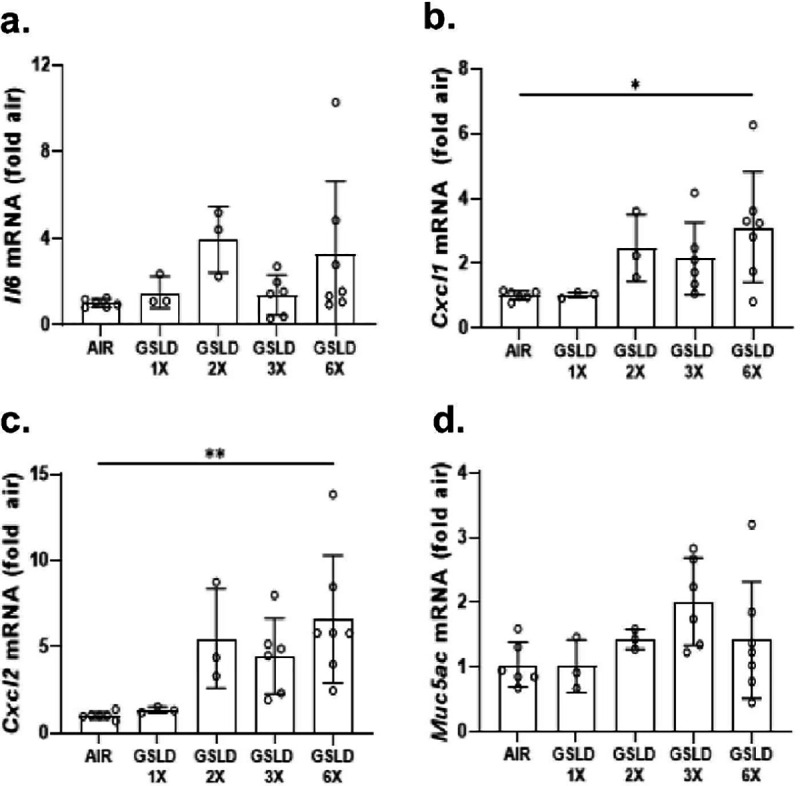
Effect of GSLD PM_2.5_ inhalation dose and duration on the expression of mRNA for cytokine and pro-inflammatory gene mRNA. (a) *Il6* (b) *Cxcl1* (c) *Cxcl2* and (d) *Muc5ac* mRNA abundance as a function of GSLD PM_2.5_ exposure. GSLD PM_2.5_ 1x and 2x (n=3). Filtered air, 3x and 6x (n=6). *p<0.05 and **p<0.01 using 1-way ANOVA and Dunnett post-test.

Mice exposed to or treated with GSLD PM_2.5_ by inhalation (Fig. 5a and b and Supplemental Fig. 5) and OPA (Supplemental Fig. 6a-f) exhibited dose-dependent increases neutrophils in BAL fluid collected 24h after treatment. Histological analysis of post-lavage tissue from both exposure paradigms confirmed pulmonary inflammation, characterized by an accumulation of neutrophils and alveolar edema (Figs. 5c–f). Neutrophilia (Ly6G^+^), changes in eosinophils, alveolar, interstitial, and monocyte-derived macrophages, CD3^+^ T cells, CD8^+^ T cells, and B cells were also shown using tissue flow cytometry (Supplemental Fig. 7). At the molecular level, both inhaled (Fig. 6a–d) and OPA-delivered GSLD PM_2.5_ (Supplemental Fig. 8a-d) induced the expression of mRNA for *Cxcl1, Cxcl2, Il6* and *Muc5ac* in mouse lungs. The effects of GSLD were dose dependent and no differences were observed between male and female mice. Using the 3x exposure paradigm, GSLD PM_2.5_ was more pro-inflammatory than an equivalent dose of CFA PM_2.5_ using neutrophilia and cytokine gene induction as the endpoints (Supplemental Fig. 9). In all cases, there was no evidence of lung infection.

### GSLD was acutely cytotoxic to HBEC3-KT and THP1 cells

**Figure 7:**
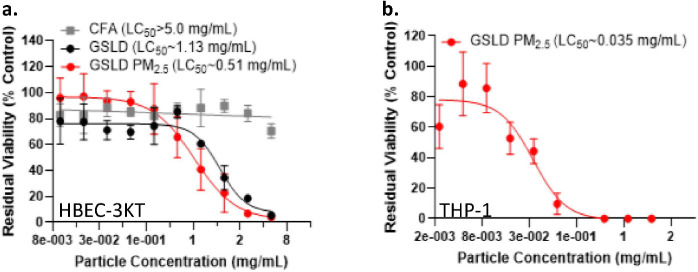
Cytotoxic potential of GSLD. (a) *In vitro* acute cytotoxicity data in HBEC3-KT cells comparing CFA, unfractionated GSL sediment, and GSLD PM_2.5_. (c) Acute cytotoxicity of GSLD PM_2.5_ in THP1 cells. A four-parameter non-linear fit was used for both datasets to estimate the LC_50_ values provided in the figures.

The acute cytotoxic effects of unfractionated GSLD and GSLD PM_2.5_ were compared to CFA in HBEC3-KT cells (Fig. 7a). GSLD was more cytotoxic than CFA; the LC_50_ for unfractionated and GSLD PM_2.5_ were 1.13 mg/mL (710 μg/cm^2^) and 0.51 mg/mL (320 μg/cm^2^), respectively. The LC_50_ for CFA was > 5 mg/mL (3,100 μg/cm^2^). The LC_50_ for GSLD using THP1 cells was ~ 0.035 mg/mL (25 μg/cm^2^; Fig. 7b)

### Mechanisms of *IL6* and *IL8* mRNA in HBEC3-KT

**Figure 8:**
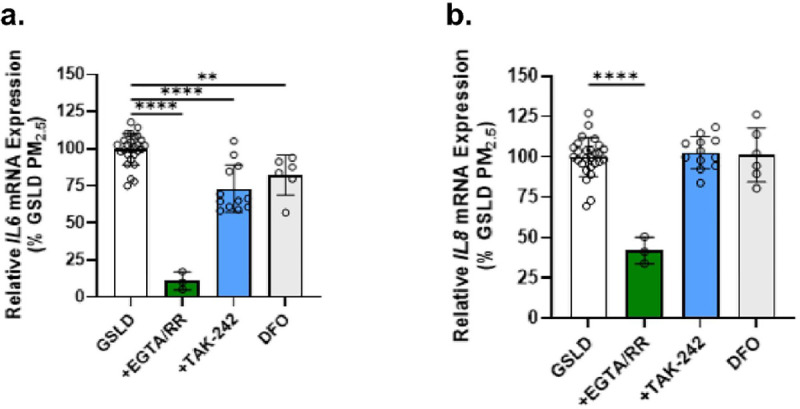
Effects of metal chelators, a calcium channel blocker, and TLR4 inhibition on *IL6/8* mRNA induction in HBEC3-KT cells. Expression of (a) *IL6* and (b) *IL8* mRNA by HBEC3-KT cells treated for 24h with GSLD PM_2.5_ (66 mg/cm^2^). The effects of co-treatment with EGTA+ruthenium red (10+50 mM; green), the metal the TLR4 inhibitor TAK-242 (1 mM; blue), and the iron chelator deferoxamine (DFO; 50μM) are shown. All treatment groups were corrected for the respective controls and normalized to GSLD PM_2.5_ treated cells. Data were analyzed using 1-way ANOVA and Dunnett post-test. **p<0.01 and ****p<0.0001.

Treatment of HBEC3-KT for 24h with GSLD PM_2.5_ (0.25 mg/mL; 66 μg/cm^2^) replicated the induction of *IL6* and *IL8* mRNA observed in mice; *MUC5AC* induction was negligible and variable (not shown). Induction of *IL6* and *8* mRNA was significantly reduced by co-treating cells with a combination of the metal/calcium chelator EGTA and the non-selective calcium channel blocker ruthenium red (Fig. 8a). The TLR4 inhibitor TAK-242 and the iron chelator deferoxamine also reduced *IL6*, but not *IL8* induction (Fig. 8b). Finally, *IL6/8* induction was not reduced by heating the GSLD PM_2.5_ to 500°C for 4h or by water and ethanol extraction.

### GSLD PM_2.5_ activated human and mouse TRP channels in HBEC3-KT cells

**Figure 9:**
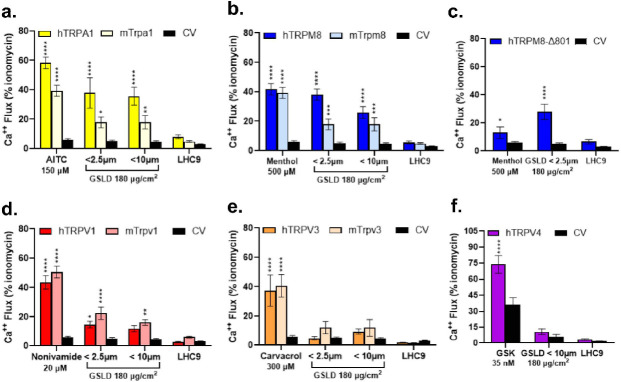
Activation of TRP ion channels by GSLD. (a-f) Normalized (to ionomycin, 10 mM) calcium flux from HEK-293 cells transiently transfected with a control (empty) or human and mouse TRP expression plasmids following treatment with GSLD. Known agonists of each channel are shown on the left side of each panel and included TRPA1 (AITC; 150μM), TRPM8 and ΔM801 (menthol; 500μM), TRPV1 (nonivamide; 20μM), and TRPV4 (GSK1016790A; 35nM). Effects of LHC9 (negative control) are on the right side of each panel. Results were compared to the control vector for each treatment using 2-way ANOVA and a Dunnett post-test. *p<0.05, **p<0.01, ***p<0.001, ****p<0.0001.

Activation of TRP family calcium channels has previously been demonstrated for CFA and other PM, and associated with cytokine gene induction and cytotoxicity in lung cells/lungs.(26, 40, 47) Activation of TRP channels by GSLD PM_10_ and PM_2.5_ was quantified using calcium flux assays. Activation of mouse (m) and human (h) TRPA1, TRPM8, the truncated TRPM8 ΔM801 variant expressed by human lung airway epithelial cells (26, 50), and TRPV1 occurred (Fig. 9a–f). TRPV3 and TRPV4 activation was not observed. Additionally, activation of mTRPA1, m/hTRPM8, and m/h TRPV1 was observed using SSD PM_2.5_ (Supplemental Fig. 10a-c).

### TRPV1 and V3 regulated *IL6/8* mRNA induction in HBEC3-KT, but not THP1 cells

**Figure 10:**
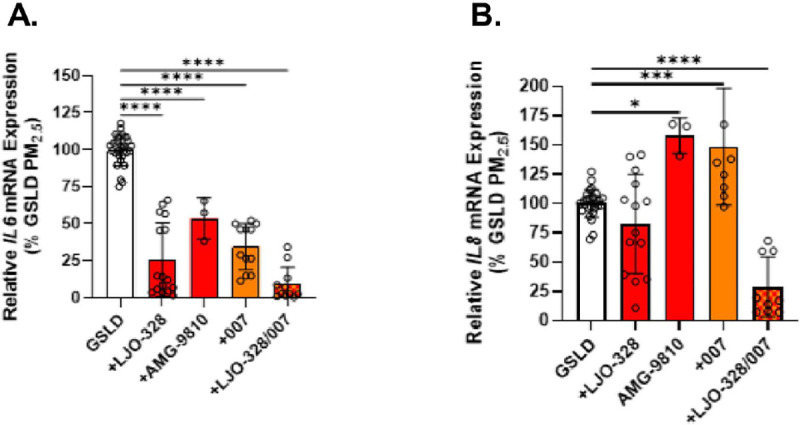
Effects of TRP channel inhibitors on *IL6/8* mRNA induction in HBEC3-KT cells. Expression of (a) *IL6* and (b) *IL8* mRNA by HBEC3-KT cells treated for 24h with GSLD PM_2.5_ (66 mg/cm^2^). The effects of co-treatment with the TRPV1 inhibitors LJO-328 and AMG-9810 (20 mM and 1 mM; red), the TRPV3 inhibitor 007 (50 mM; orange), and a combination of LJO-328 and 007 (20+50μM; red/orange checkered) are shown. All treatment groups were corrected for the respective controls and normalized to GSLD PM_2.5_ treated cells. Data were analyzed using 1-way ANOVA and Dunnett post-test. *p<0.05, ***p<0.001, and ****p<0.0001.

The effects of pre- and co-treatment of HBEC3-KT cells with GSLD PM_2.5_ and TRP channel antagonists was screened using *IL6* and *IL8* mRNA induction as an endpoint (Supplemental Fig. 11a and b). Inhibitors of TRPA1 (A967079), TRPM8/TRPM8 ΔM801 (AMTB), and TRPV4 (HC067047) did not affect mRNA induction, while inhibition of TRPV1 (LJO-328) and TRPV3 (007) showed variable effects, as a function of gene (*IL6* was attenuated by both, but *IL8* was increased by 007). Subsequent experiments confirmed the inhibition of *IL6* induction by LJO-328, AMG-9810 (another TRPV1 antagonist), and a combination of LJO-328 and 007, with the latter showing an additive effect (Fig. 10a). As above, variable effects were observed for *IL8* induction, where both TRPV1 and V3 inhibition appeared to increase *IL8* induction (Fig. 10b). However, the combination of LJO-328 and 007 was inhibitory. Similar inhibition of IL6 was observed using SSD (Supplemental Figs. 11a and b). Finally, induction of mRNA for *IL6, IL8*, and *TNFa* was also evaluated in THP1 cells. Induction of *IL6*, but not *IL8* or *TNFa* mRNA by LPS was reduced ~ 95% by TAK-242 (p < 0.0001), and ~ 40% (p < 0.001) and ~ 25% (not significant) for GSLD and SSD PM_2.5_, respectively (Supplemental Figs. 11c). TRPV1 and V3 inhibition had no effect on the responses of the THP1 cells to GSLD PM_2.5_.

### Humanized Trpv1^N606D^ mice exhibited greater neutrophilia than wild-type mice:

**Figure 11:**
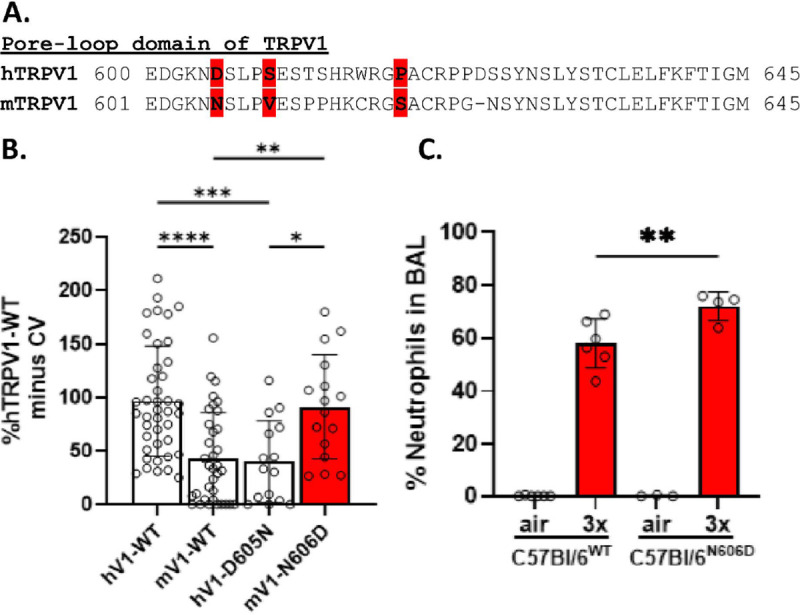
“Humanization” of mouse TRPV1 increased BAL neutrophils following GSLD PM_2.5_ inhalation. (a) Comparison of the amino acid sequence of the pore-loop region (residues 600/601–645) of human (h) and mouse (m) TRPV1. (b) Calcium flux data using transfected HEK-293 cells transiently transfected with expression plasmids harboring human and mouse TRPV1, with and without mutations of residues 605/606 to the corresponding human and mouse residues. CFA (180 mg/cm^2^) was used as the agonist and mouse TRPV1 N606D is shown in red. (c) comparison of BAL neutrophils in wild-type C57Bl/6 and *Trpv1*^N606D^ “humanized” mice (50 mg/m^3^; 3x exposure paradigm). N≥3, p<0.05, **p<0.01, and ***p<0.001 using

**M**ice were created based on the hypothesis and *in vitro* calcium assay results showing that mouse TRPV1 was less sensitive to CFA PM but became sensitized by mutation of N606 to the corresponding human residue (D), while converting human D605 to the mouse residue decreased activation by CFA (Fig. 11A and B). Using the 3x inhalation exposure paradigm, Trpv1^N606D^ mice exhibited increased (~ 15%) neutrophilia compared to wild-type controls (Fig. 11C), supporting a role for TRPV1 in initiating lung inflammation in the respiratory tract with GSLD PM_2.5_ inhalation.

## Discussion

This study shows that GSLD and regional dusts contribute to PM_2.5_ and PM_10_ pollution in the Salt Lake Valley and Wasatch front during high wind events. There are ~ 2.5 M people residing in the Salt Lake Valley/Wasatch front and PM concentrations during dust events can temporarily exceed levels defined as unhealthy for sensitive individuals (PM_2.5_ 35.5–55.4 μg/m^3^), and in some locations and events, even healthy individuals (> 55.4 μg/m^3^), based on current EPA and Utah Department of Air Quality standards. If the GSL were to continue to lose water, additional sediments would become exposed, potentially increasing dust burden in the local airshed.(51) It is therefore critical to understand the potential health risks posed by inhalation and other exposures to GSLD. This study is the first to evaluate the acute effects of inhaled GSLD PM_2.5_, and while cursory, provides the first evidence that respirable GSLD could adversely impact pulmonary and presumably other aspects of human health among people living along the Wasatch Front in Utah, including Salt Lake City and other major cities (e.g., Ogden and Provo).

In mice, neutrophilia and edema were the principal effects elicited by GSLD PM_2.5_, indicating an acute inflammatory response. Consistent with this effect and known biological activities, increased expression of mRNA for *Il6, Cxcl1, Cxcl2* was also observed.(52, 53) Additionally, *Muc5ac* RNA was induced, which can contribute to airway obstruction and hypersensitivity in asthma.(54–57) These responses are normal and occur in response to a variety of pathogens and other foreign materials entering the respiratory tract, including other forms of PM. In the average healthy person, these effects may go unnoticed and resolve quickly when pollution levels return to baseline levels. However, in some individuals, such as those with asthma and other pre-existing conditions including COPD, heart disease, and obesity/metabolic syndrome, acute lung inflammation could be amplified, slower to resolve, and exacerbate the underlying conditions leading to more serious adverse sequela including increased risks for hospitalization and even death. Children and the elderly are also at greater risk, and the consequences of lung inflammation and injury early in life are recognized as a risk factor for poor respiratory health later in life.(58, 59)The fact that GSLD is able to elicit inflammation at these environmentally relevant concentrations and doses, and that the effects were substantially greater than those of a known pneumotoxic PM, CFA, supports the notion that GSLD could be a threat to the well-being of local residents.

Because the health effects and effects of PM can vary as a function of dose and particle composition, among other human factors (e.g., genetics), the composition of GSLD PM_2.5_ was partially elucidated and key facets systematically tested. Of concern, GSL sediments and GSLD PM_2.5_ contained multiple known human toxins including LPS, organic pollutants of natural and anthropogenic origin, redox active transition metals (iron, copper) and other toxic metals (arsenic, lead, etc.), as well as pathogenic bacteria (*Streptococcus pyogenes*). LPS/endotoxin is a potent pneumotoxin that triggers neutrophilia and edema,(60) and intranasal and systemically administered LPS are used as models for septic lung injury.(61) Indeed, TLR4 inhibition using TAK-242 slightly reduced the pro-inflammatory responses of HBEC3-KT cells, principally the induction of IL6, which is a crucial acute phase response protein that regulates inflammation and impacts health/diseases, including asthma.(62–64) Thus, modulation of by GSLD and various inhibitors IL6 is noteworthy. While we did not observe lung infection, the potential also exists and should not be overlooked as a potential risk that could also impact people exposed to GSLD.

Redox active transition metals such as iron and copper are essential, but also acutely and chronically toxic in the lungs by virtue of their ability to form insoluble aggregates (e.g. ferruginous bodies) that catalyze free radical reactions that cause oxidative stress and permanent cell/tissue injury.(65) Additionally, it has been shown that PM containing metals and organic chemicals form environmentally persistent free radicals which contribute to adverse respiratory and cardiovascular effects.(66) Results using EGTA and deferoxamine support a role for metals, presumably iron associated with GSLD, in mediating inflammatory responses in HBEC3-KT cells, albeit the relative importance appeared to be limited. Regardless, the presence of both redox-active and known toxic metals in GSLD is significant with respect to both the acute and potential long-term risks of exposure to GSLD.

Organic chemicals arising from natural and anthropogenic sources were also preliminarily identified in GSLD. Results indicated numerous chemicals representing human metabolites of common therapeutics and hormones, and industrial and agricultural chemicals were present in extracts of GSLD. While the presence and concentrations of the various compounds were not validated or quantified, it is possible that one or more of these agents could pose a risk to human health and contribute to the overall effects of GSLD. An example is the identification of the neurotoxin β-methylamino-L-alanine in GSLD samples by Piotrowicz *et al*. (unpublished). While neither heating nor extraction of the GSLD reduced the pro-inflammatory potential of GSLD PM_2.5_ using HBEC3-KT cells, more comprehensive studies evaluating the impact of chemical pollutants, in addition to the PM itself, should also be pursued.

Overall, there is a relative paucity of information regarding the effects of PM originating from geological and other “natural sources” such as GSLD when compared to PM derived from burning biomass, fossil fuels, etc., which are widely recognized as unhealthy. Previous work by our group demonstrated that soil dusts are in fact pro-inflammatory and cytotoxic to lung cells, but in general, less “toxic” compared to commonly studied emission particles.(67–69) Here, we compared the effects of inhaled GSLD PM_2.5_ to those of CFA PM_2.5_. CFA is a combustion by-product from coal burning power plants that is similar in elemental composition to GSLD in that it is a calcium/magnesium/aluminum-rich particle that promotes inflammatory cytokine production in human lung epithelial cells and in mice, in part via activation of TRPV1,(40) TRPM8,(26) and TRPA1.(70) Interestingly, but perhaps not surprisingly, GSLD PM_2.5_ was substantially more cytotoxic to human lung cells *in vitro*, and more pro-inflammatory in mice. Meanwhile, another saline lake dust, SSD, for which adverse human health effects have been described,(14, 15, 17, 18, 20, 21) exhibited overlapping activities with, and seemingly greater potency than GSLD. Remarkably, both GSLD and SSD appeared to engage TRPV1 and TRPV3 as a basis for stimulating *IL6* and *IL8* expression by HBEC3-KT cells, intimating the potential of perhaps therapeutically modulating these receptors as a way to mitigate the acute and possibly longer-term effects of GSLD and similar saline lake dusts. While highly speculative, the idea of targeting at least TRPV1 is supported by results using humanized TRPV1 mice, which imply that TRPV1 activation is at least in part responsible for lung inflammation elicited by GSLD.

## Conclusions

This study highlights potential risks associated with GSLD inhalation and suggests that the effects of GSLD may be modeled by and a model for other saline lake dusts for which health effects have been described. Thus, the potential of GSLD and related saline or even non-saline lake dusts to cause adverse health outcomes should be explored in greater depth in order to understand the breadth of effects and risks that these materials have on people living in areas prone to dust events, and to further develop the mechanistic knowledge needed to design effective interventional strategies to protect people at risk for developing acute and potentially long-term adverse effects. Finally, this work supports current efforts to develop aggressive conservation measures to protect the GSL to prevent further loss of water.

## Data Availability

All data generated or analyzed during this study are included in this published article and its supplementary information files and are available from the authors upon reasonable request.

## References

[1] Benson LV, Lund SP, Smoot JP, Rhode DE, Spencer RJ, Verosub KL The rise and fall of Lake Bonneville between 45 and 10.5 ka. 2011;235:57–69.

[2] Spencer RJ, Baedecker MJ, Eugster HP, Forester RM, Goldhaber MB, Jones BF Great Salt Lake, and precursors, Utah: The last 30,000 years. 1984;86:321 – 34.

[3] Naftz D, Angeroth C, Kenney T, Waddell B, Darnall N, Silva S Anthropogenic influences on the input and biogeochemical cycling of nutrients and mercury in Great Salt Lake, Utah, USA. 2008;23:1731–44.

[4] Wurtsbaugh WA, Leavitt PR, Moser KA. Effects of a century of mining and industrial production on metal contamination of a model saline ecosystem. Great Salt Lake Utah. 2020;266:115072. 10.1016/j.envpol.2020.115072 32836014

[5] Baxter BK. Great Salt Lake microbiology: a historical perspective. 2018;21:79–95. 10.1007/s10123-018-0008-z PMC6133049 30810951

[6] Davis J, Gwynn JW, Rupke A. Commonly Asked Questions About Utah’s Great Salt Lake and Ancient Lake Bonneville. 2022.

[7] Isaacson AE, Hachman F, Robson R. The economics of Great Salt Lake. Great Salt Lake: an overview of change. 2002:187–200.

[8] Bioeconomics I. Economic Significance of the Great Salt Lake to the State of Utah. 2012.

[9] AghaKouchak A, Norouzi H, Madani K, Mirchi A, Azarderakhsh M, Nazemi A Aral Sea syndrome desiccates Lake Urmia: Call for action. 2015;41:307 – 11.

[10] Wurtsbaugh WA, Miller C, Null SE, DeRose RJ, Wilcock P, Hahnenberger M, Decline world’s saline lakes. 2017;10:816–21.

[11] Micklin P. Aral Sea Disaster. 2007;35:47–72.

[12] Crighton EJ, Barwin L, Small I, Upshur R. What have we learned? A review of the literature on children’s health and the environment in the Aral Sea area. Int J Public Health. 2011;56(2):125–38. 20976516 10.1007/s00038-010-0201-0 PMC3066395

[13] Feizizadeh B, Lakes T, Omarzadeh D, Pourmoradian S. Health effects of shrinking hyper-saline lakes: spatiotemporal modeling of the Lake Urmia drought on the local population, case study of the Shabestar County. 2023;13:1622. 10.1038/s41598-023-28332-6 PMC9884074 36709338

[14] Biddle TA, Li Q, Maltz MR, Tandel PN, Chakraborty R, Yisrael K, Salton Sea aerosol exposure in mice induces a pulmonary response distinct from allergic inflammation. Sci Total Environ. 2021;792:148450. 34157526 10.1016/j.scitotenv.2021.148450

[15] Burr AC, Velazquez JV, Ulu A, Kamath R, Kim SY, Bilg AK, Lung Inflammatory Response to Environmental Dust Exposure in Mice Suggests a Link to Regional Respiratory Disease Risk. J Inflamm Res. 2021;14:4035–52. 34456580 10.2147/JIR.S320096 PMC8387588

[16] D’Evelyn SM, Vogel C, Bein KJ, Lara B, Laing EA, Abarca RA Differential inflammatory potential of particulate matter (PM) size fractions from Imperial Valley, CA. Atmos Environ (1994). 2021;244. 10.1016/j.atmosenv.2020.117992 PMC7654835 33184556

[17] Frie AL, Dingle JH, Ying SC, Bahreini R. The Effect of a Receding Saline Lake (The Salton Sea) on Airborne Particulate Matter Composition. Environ Sci Technol. 2017;51(15):8283–92. 28697595 10.1021/acs.est.7b01773

[18] Johnston JE, Razafy M, Lugo H, Olmedo L, Farzan SF. The disappearing Salton Sea: A critical reflection on the emerging environmental threat of disappearing saline lakes and potential impacts on children’s health. Sci Total Environ. 2019;663:804–17. 30738261 10.1016/j.scitotenv.2019.01.365 PMC7232737

[19] Kittle S. Survey of Reported Health Effects of Owens Lake Particulate Matter 2000.

[20] Marshall JR. Why Emergency Physicians Should Care About the Salton Sea. West J Emerg Med. 2017;18(6):1008–9. 29085530 10.5811/westjem.2017.8.36034 PMC5654867

[21] Miao Y, Porter WC, Schwabe K, LeComte-Hinely J. Evaluating health outcome metrics and their connections to air pollution and vulnerability in Southern California’s Coachella Valley. Sci Total Environ. 2022;821:153255. 35066029 10.1016/j.scitotenv.2022.153255

[22] Carling GT, Fernandez DP, Rey KA, Hale CA, Goodman MM, Nelson ST. Using strontium isotopes to trace dust from a drying Great Salt Lake to adjacent urban areas and mountain snowpack. 2020;15:114035.

[23] Lang OI, Mallia D, Skiles SM. The shrinking Great Salt Lake contributes to record high dust-on-snow deposition in the Wasatch Mountains during the 2022 snowmelt season. Environ Res Lett. 2023;18(6).

[24] Jones BA, Fleck J. Shrinking lakes, air pollution, and human health: Evidence from California’s Salton Sea. Sci Total Environ. 2020;712:136490. 31931219 10.1016/j.scitotenv.2019.136490

[25] Deering-Rice CE, Mitchell VK, Romero EG, Abdel Aziz MH, Ryskamp DA, Krizaj D, Drofenine: A 2-APB Analogue with Greater Selectivity for Human TRPV3. Pharmacol Res Perspect. 2014;2(5):e00062. 25089200 10.1002/prp2.62 PMC4115637

[26] Lamb JG, Romero EG, Lu ZY, Marcus SK, Peterson HC, Veranth JM, Activation of Human Transient Receptor Potential Melastatin-8 (TRPM8) by Calcium-Rich Particulate Materials and Effects on Human Lung Cells. Mol Pharmacol. 2017;92(6):653–64. 29038158 10.1124/mol.117.109959 PMC5695664

[27] Smith KR, Veranth JM, Kodavanti UP, Aust AE, Pinkerton KE. Acute pulmonary and systemic effects of inhaled coal fly ash in rats: comparison to ambient environmental particles. Toxicol Sci. 2006;93(2):390–9. 16840564 10.1093/toxsci/kfl062

[28] Veranth JM, Smith KR, Huggins F, Hu AA, Lighty JS, Aust AE. Mossbauer spectroscopy indicates that iron in an aluminosilicate glass phase is the source of the bioavailable iron from coal fly ash. Chem Res Toxicol. 2000;13(3):161–4. 10725111 10.1021/tx9902136

[29] Becnel T, Tingey K, Whitaker J, Sayahi T, Le K, Goffin P, A Distributed Low-Cost Pollution Monitoring Platform. Ieee Internet Things. 2019;6(6):10738–48.

[30] Kelly KE, Whitaker J, Petty A, Widmer C, Dybwad A, Sleeth D, Ambient and laboratory evaluation of a low-cost particulate matter sensor. Environ Pollut. 2017;221:491–500. 28012666 10.1016/j.envpol.2016.12.039 PMC10625486

[31] Mallia DV, Kochanski AK, Kelly KE, Whitaker R, Xing W, Mitchell LE, Evaluating Wildfire Smoke Transport Within a Coupled Fire-Atmosphere Model Using a High-Density Observation Network for an Episodic Smoke Event Along Utah’s Wasatch Front. J Geophys Research: Atmos. 2020;125(20):e2020JD032712.

[32] Sayahi T, Butterfield A, Kelly KE. Long-term field evaluation of the Plantower PMS low-cost particulate matter sensors. Environ Pollut. 2019;245:932–40. 30682749 10.1016/j.envpol.2018.11.065

[33] Sayahi T, Kaufman D, Becnel T, Kaur K, Butterfield AE, Collingwood S, Development of a calibration chamber to evaluate the performance of low-cost particulate matter sensors. Environ Pollut. 2019;255(Pt 1):113131. 31521992 10.1016/j.envpol.2019.113131 PMC7409587

[34] Kaur KaK KE. Performance evaluation of the Alphasense OPC-N3 and Plantower PMS5003 sensor in measuring dust events in the Salt Lake Valley, Utah. Atmos Meas Tech. 2023;16(10):2455–70.

[35] Wang M, Carver JJ, Phelan VV, Sanchez LM, Garg N, Peng Y, Sharing and community curation of mass spectrometry data with Global Natural Products Social Molecular Networking. Nat Biotechnol. 2016;34(8):828–37. 27504778 10.1038/nbt.3597 PMC5321674

[36] Memon TA, Sun L, Almestica-Roberts M, Deering-Rice CE, Moos PJ, Reilly CA. Inhibition of TRPA1, Endoplasmic Reticulum Stress, Human Airway Epithelial Cell Damage, and Ectopic MUC5AC Expression by Vasaka (Adhatoda vasica; Malabar Nut) Tea. Pharmaceuticals (Basel). 2023;16(6). 10.3390/ph16060890 PMC10303053 37375837

[37] Borghi F, Spinazze A, Mandaglio S, Fanti G, Campagnolo D, Rovelli S Estimation of the Inhaled Dose of Pollutants in Different Micro-Environments: A Systematic Review of the Literature. Toxics. 2021;9(6). 10.3390/toxics9060140 PMC8231583 34204794

[38] Schwarte LA, Zuurbier CJ, Ince C. Mechanical ventilation of mice. Basic Res Cardiol. 2000;95(6):510–20. 11192374 10.1007/s003950070029 PMC7102075

[39] Kolanjiyil AV, Kleinstreuer C, Kleinstreuer NC, Pham W, Sadikot RT. Mice-to-men comparison of inhaled drug-aerosol deposition and clearance. Respir Physiol Neurobiol. 2019;260:82–94. 30445230 10.1016/j.resp.2018.11.003

[40] Deering-Rice CE, Johansen ME, Roberts JK, Thomas KC, Romero EG, Lee J Transient Receptor Potential Vanilloid-1 (TRPV1) Is a Mediator of Lung Toxicity for Coal Fly Ash Particulate Material. 2012;81:411–9. 10.1124/mol.111.076067 PMC3286291 22155782

[41] Deering-Rice CE, Nguyen N, Lu Z, Cox JE, Shapiro D, Romero EG, Activation of TRPV3 by Wood Smoke Particles and Roles in Pneumotoxicity. Chem Res Toxicol. 2018;31(5):291–301. 29658714 10.1021/acs.chemrestox.7b00336 PMC6342208

[42] Kim YH, Warren SH, Kooter I, Williams WC, George IJ, Vance SA, Chemistry, lung toxicity and mutagenicity of burn pit smoke-related particulate matter. Part Fibre Toxicol. 2021;18(1):45. 34915899 10.1186/s12989-021-00435-w PMC8675519

[43] Bonner JC, Silva RM, Taylor AJ, Brown JM, Hilderbrand SC, Castranova V, Interlaboratory evaluation of rodent pulmonary responses to engineered nanomaterials: the NIEHS Nano GO Consortium. Environ Health Perspect. 2013;121(6):676–82. 23649427 10.1289/ehp.1205693 PMC3672912

[44] Kim YH, Vance SA, Aurell J, Holder AL, Pancras JP, Gullett B, Chemistry and lung toxicity of particulate matter emitted from firearms. Sci Rep. 2022;12(1):20722. 36456643 10.1038/s41598-022-24856-5 PMC9715551

[45] Zychowski KE, Kodali V, Harmon M, Tyler CR, Sanchez B, Ordonez Suarez Y, Respirable Uranyl-Vanadate-Containing Particulate Matter Derived From a Legacy Uranium Mine Site Exhibits Potentiated Cardiopulmonary Toxicity. Toxicol Sci. 2018;164(1):101–14. 29660078 10.1093/toxsci/kfy064 PMC6016706

[46] Nguyen J, Deering-Rice CE, Armstrong BS, Massa C, Reilly CA, Venosa A. Parenchymal and Inflammatory Cell Responses to Single and Repeated Ozone Exposure in Healthy and Surfactant Protein-C Mutant Lung. Toxicol Sci. 2022;189(1):107–23. 35866636 10.1093/toxsci/kfac074 PMC9412175

[47] Deering-Rice CE, Romero EG, Shapiro D, Hughen RW, Light AR, Yost GS, Electrophilic Components of Diesel Exhaust Particles (DEP) Activate Transient Receptor Potential Ankyrin-1 (TRPA1): A Probable Mechanism of Acute Pulmonary Toxicity for DEP. Chem Res Toxicol. 2011;24(6):950–9. 21591660 10.1021/tx200123z PMC3133601

[48] FOX 13 News. Blowing dust causes dirty rain to fall across northern Utah 2023 [ https://www.fox13now.com/weather/blowing-dust-causes-dirty-rain-to-fall-across-northern-utah#:~:text=%22Muddy%20rain%22%20has%20been%20falling,else%20exposed%20to%20the%20weather.

[49] Bring Horvath ER, Brazelton WJ, Kim MC, Cullum R, Mulvey MA, Fenical W, Bacterial diversity and chemical ecology of natural product-producing bacteria from Great Salt Lake sediment. ISME Commun. 2024;4(1):ycae029. 38524762 10.1093/ismeco/ycae029 PMC10960970

[50] Sabnis AS, Reilly CA, Veranth JM, Yost GS. Increased transcription of cytokine genes in human lung epithelial cells through activation of a TRPM8 variant by cold temperatures. Am J Physiol Lung Cell Mol Physiol. 2008;295(1):L194–200. 18441098 10.1152/ajplung.00072.2008 PMC2491334

[51] Hahnenberger M, Nicoll K. Meteorological characteristics of dust storm events in the eastern Great Basin of Utah, USA. Atmos Environ. 2012;60:601–12.

[52] Sawant KV, Sepuru KM, Lowry E, Penaranda B, Frevert CW, Garofalo RP, Neutrophil recruitment by chemokines Cxcl1/KC and Cxcl2/MIP2: Role of Cxcr2 activation and glycosaminoglycan interactions. J Leukoc Biol. 2021;109(4):777–91. 32881070 10.1002/JLB.3A0820-207R PMC8296306

[53] Yang J, Chen Y, Yu Z, Ding H, Ma ZF. The influence of PM2.5 on lung injury and cytokines in mice. Exp Ther Med. 2019;18(4):2503–11. 31572502 10.3892/etm.2019.7839 PMC6755482

[54] Caramori G, Casolari P, Di Gregorio C, Saetta M, Baraldo S, Boschetto P, MUC5AC expression is increased in bronchial submucosal glands of stable COPD patients. Histopathology. 2009;55(3):321–31. 19723147 10.1111/j.1365-2559.2009.03377.x

[55] Conti C, Montero-Fernandez A, Borg E, Osadolor T, Viola P, De Lauretis A, Mucins MUC5B and MUC5AC in Distal Airways and Honeycomb Spaces: Comparison among Idiopathic Pulmonary Fibrosis/Usual Interstitial Pneumonia, Fibrotic Nonspecific Interstitial Pneumonitis, and Control Lungs. Am J Respir Crit Care Med. 2016;193(4):462–4. 26871672 10.1164/rccm.201507-1322LE

[56] Evans CM, Raclawska DS, Ttofali F, Liptzin DR, Fletcher AA, Harper DN, The polymeric mucin Muc5ac is required for allergic airway hyperreactivity. Nat Commun. 2015;6:6281. 25687754 10.1038/ncomms7281 PMC4333679

[57] Livraghi-Butrico A, Grubb BR, Wilkinson KJ, Volmer AS, Burns KA, Evans CM, Contribution of mucus concentration and secreted mucins Muc5ac and Muc5b to the pathogenesis of muco-obstructive lung disease. Mucosal Immunol. 2017;10(3):829. 10.1038/mi.2017.29 28435155

[58] Bobolea I, Arismendi E, Valero A, Agusti A. Early Life Origins of Asthma: A Review of Potential Effectors. J Investig Allergol Clin Immunol. 2019;29(3):168–79. 10.18176/jiaci.0361 30561365

[59] Grant T, Brigham EP, McCormack MC. Childhood Origins of Adult Lung Disease as Opportunities for Prevention. J Allergy Clin Immunol Pract. 2020;8(3):849–58. 32147138 10.1016/j.jaip.2020.01.015 PMC7653544

[60] Horgan MJ, Palace GP, Everitt JE, Malik AB. Tnf-Alpha Release in Endotoxemia Contributes to Neutrophil-Dependent Pulmonary-Edema. Am J Physiol. 1993;264(4):H1161–5. 8476094 10.1152/ajpheart.1993.264.4.H1161

[61] Matute-Bello G, Frevert CW, Martin TR. Animal models of acute lung injury. Am J Physiol Lung Cell Mol Physiol. 2008;295(3):L379–99. 18621912 10.1152/ajplung.00010.2008 PMC2536793

[62] Tanaka T, Narazaki M, Kishimoto T. IL-6 in inflammation, immunity, and disease. Cold Spring Harb Perspect Biol. 2014;6(10):a016295. 25190079 10.1101/cshperspect.a016295 PMC4176007

[63] Peters MC, Mauger D, Ross KR, Phillips B, Gaston B, Cardet JC, Evidence for Exacerbation-Prone Asthma and Predictive Biomarkers of Exacerbation Frequency. Am J Respir Crit Care Med. 2020;202(7):973–82. 32479111 10.1164/rccm.201909-1813OC PMC7528796

[64] Rose-John S. Interleukin-6 signalling in health and disease. F1000Res. 2020;9. 32864098 10.12688/f1000research.26058.1 PMC7443778

[65] Fariss MW, Gilmour MI, Reilly CA, Liedtke W, Ghio AJ. Emerging mechanistic targets in lung injury induced by combustion-generated particles. Toxicol Sci. 2013;132(2):253–67. 23322347 10.1093/toxsci/kft001 PMC4447844

[66] Aryal A, Noel A, Khachatryan L, Cormier SA, Chowdhury PH, Penn A, Environmentally persistent free radicals: Methods for combustion generation, whole-body inhalation and assessing cardiopulmonary consequences. Environ Pollut. 2023;334:122183. 37442324 10.1016/j.envpol.2023.122183 PMC10528481

[67] Veranth JM, Cutler NS, Kaser EG, Reilly CA, Yost GS. Effects of cell type and culture media on Interleukin-6 secretion in response to environmental particles. Toxicol Vitro. 2008;22(2):498–509. 10.1016/j.tiv.2007.10.011 PMC2464571 18178371

[68] Veranth JM, Moss TA, Chow JC, Labban R, Nichols WK, Walton JC, Correlation of in vitro cytokine responses with the chemical composition of soil-derived particulate matter. Environ Health Perspect. 2006;114(3):341–9. 16507455 10.1289/ehp.8360 PMC1392226

[69] Veranth JM, Reilly CA, Veranth MM, Moss TA, Langelier CR, Lanza DL, Inflammatory cytokines and cell death in BEAS-2B lung cells treated with soil dust, lipopolysaccharide, and surface-modified particles. Toxicol Sci. 2004;82(1):88–96. 15310859 10.1093/toxsci/kfh248 PMC2292468

[70] Deering-Rice CE, Stockmann C, Romero EG, Lu Z, Shapiro D, Stone BL, Characterization of Transient Receptor Potential Vanilloid-1 (TRPV1) Variant Activation by Coal Fly Ash Particles and Associations with Altered Transient Receptor Potential Ankyrin-1 (TRPA1) Expression and Asthma. J Biol Chem. 2016;291(48):24866–79. 27758864 10.1074/jbc.M116.746156 PMC5122759

